# An Innovative Primary Repair Technique for Large Cutaneous Defects Post-excision of Complicated Preauricular Sinuses: A Case Report

**DOI:** 10.7759/cureus.64763

**Published:** 2024-07-17

**Authors:** Kuganathan Ramasamy, Noraimi Khamalrudin, Davina Stasia Hui Ming Teo, Noor Dina Hashim

**Affiliations:** 1 Otorhinolaryngology - Head and Neck Surgery, Hospital Shah Alam, Shah Alam, MYS; 2 Otorhinolaryngology - Head and Neck Surgery, Universiti Kebangsaan Malaysia Medical Centre, Kuala Lumpur, MYS

**Keywords:** tissue adhesive, wound closure, technique, reconstruction, preauricular

## Abstract

Preauricular sinuses are congenital anomalies arising from the incomplete fusion of hillocks of His of the first and second branchial arches. Surgery is warranted when there is recurrent infection or abscess formation. However, the presence of scarring and skin thinning could result in large tissue defects after complete excision. In such cases, meticulous preoperative planning with regard to the reconstruction technique is imperative. We describe the clinical presentation, surgical technique, and postoperative outcomes of such a case in a young toddler, with a focus on the rationale behind the chosen management strategy. By sharing our experience, we aim to contribute to the existing literature on the management of complicated preauricular sinuses and provide insights that may guide clinicians facing similar challenges.

## Introduction

Preauricular sinuses are congenital anomalies arising from incomplete fusion of hillocks of His of the first and second branchial arches. The true prevalence remains elusive, as it varies according to different ethnic groups from different parts of the world. A study by Huang et al. reported a prevalence ranging from 0.17% to 1.36% across the three main ethnicities in Singapore, namely Chinese, Malay, and Indian [1]. Albeit mostly asymptomatic, preauricular sinuses can be a teething problem when complicated by recurrent infections and subsequent scarring that ensues. In such cases, complete excision of the sinus is mandated to prevent further complications. However, the surgical management of such complicated preauricular sinuses presents significant reconstruction challenges [2,3]. Ensuring complete excision of the sinus while achieving optimal wound closure with minimal cosmetic deformity may not be a straightforward task. This is particularly challenging in pediatric patients, as any complications arising from the surgery might be more tedious to manage [3].

We present a case involving a young boy with a complicated preauricular sinus that required complete excision, albeit resulting in a large cutaneous defect. The challenge was to achieve complete closure of this sizable defect without compromising the aesthetic outcome. Despite the complexity of the defect, primary closure was successfully achieved with the use of topical skin adhesive containing 2-octyl cyanoacrylate (OCA) (Surgiseal®; H.B. Fuller Medical Adhesive Technologies, LLC, Hudson, NC, USA).

This case highlights the importance of thorough surgical planning and the use of innovative approaches in achieving favorable outcomes in complicated preauricular sinus cases. This approach is particularly valuable in pediatric patients, where achieving both functional and cosmetic success is paramount.

## Case presentation

**Figure 1:**
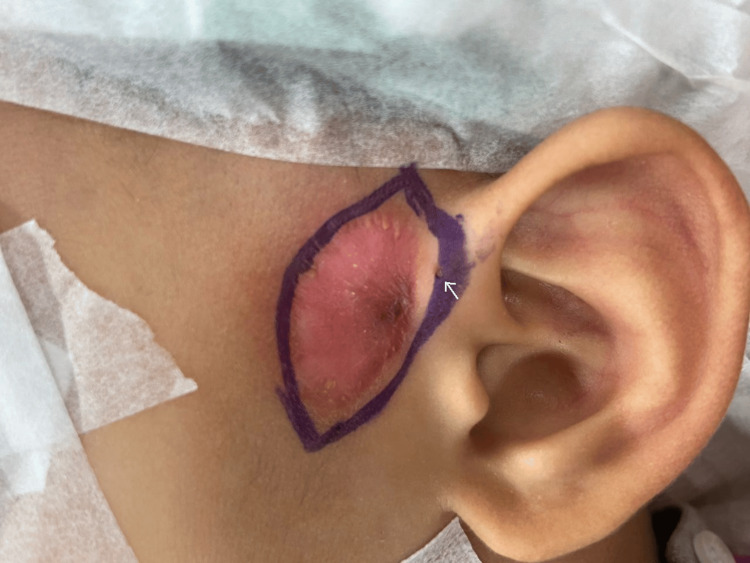
Left preauricular sinus (arrow) at the ascending limb of the helix with adjacent scar tissue measuring 3 x 2 cm

A three-year-old boy was referred to the ENT team for recurrent left preauricular sinus infection. He has had multiple episodes of infection for the past two years, including two-incision and drainage procedures in the preceding year. His parents mentioned bouts of intermittent discharges as well, apart from the conspicuous episodes of infection. The recurrent infections and abscess drainages had resulted in a 3 x 2 cm scar tissue with thin skin (Figure 1).

Surgery to remove the sinus tract and the adjacent scar tissue was proposed. The parents were counseled on the indications for surgery to prevent further infective episodes, progressive scarring, and the risk of arterial injury due to the thin overlying skin. Additionally, the potential surgical risks, including altered facial cosmesis, wound breakdown, and, rarely, facial nerve injury, were explained to them.

**Figure 2:**
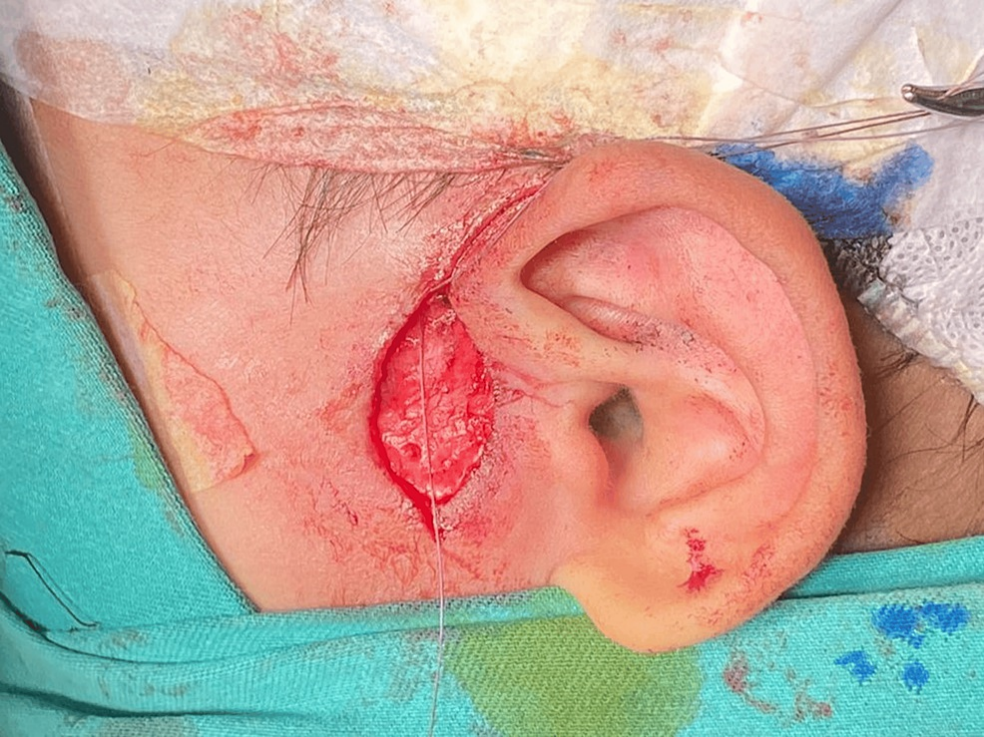
Wide-undermining of the wound edges performed

**Figure 3:**
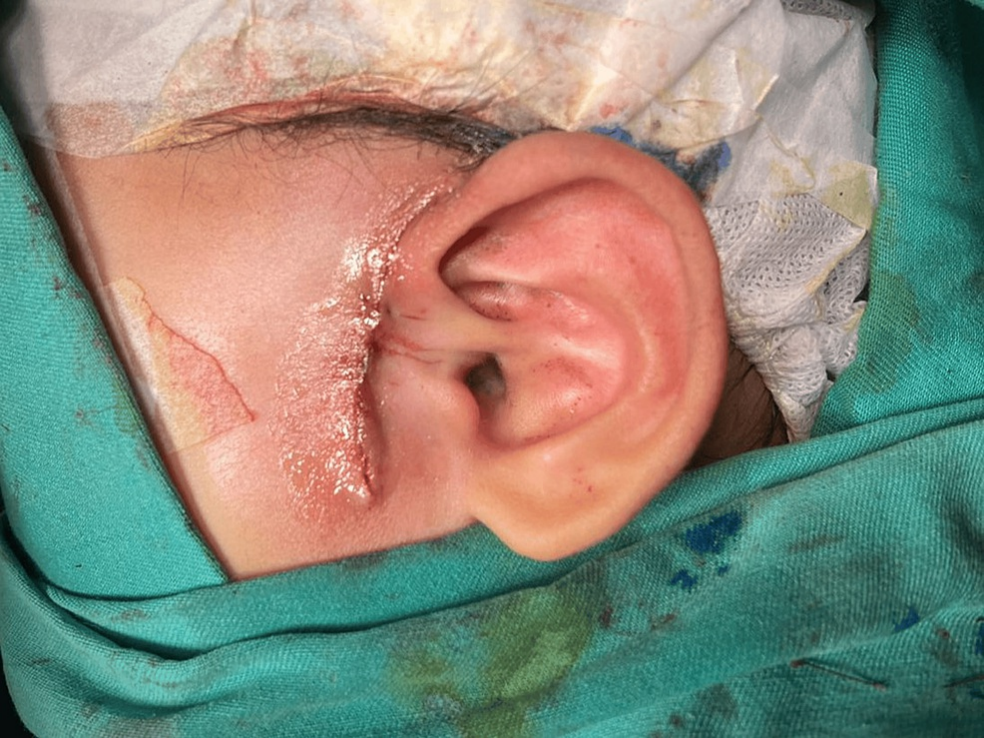
Subcutaneous layer closure performed followed by application of Surgiseal® topical skin adhesive

**Figure 4:**
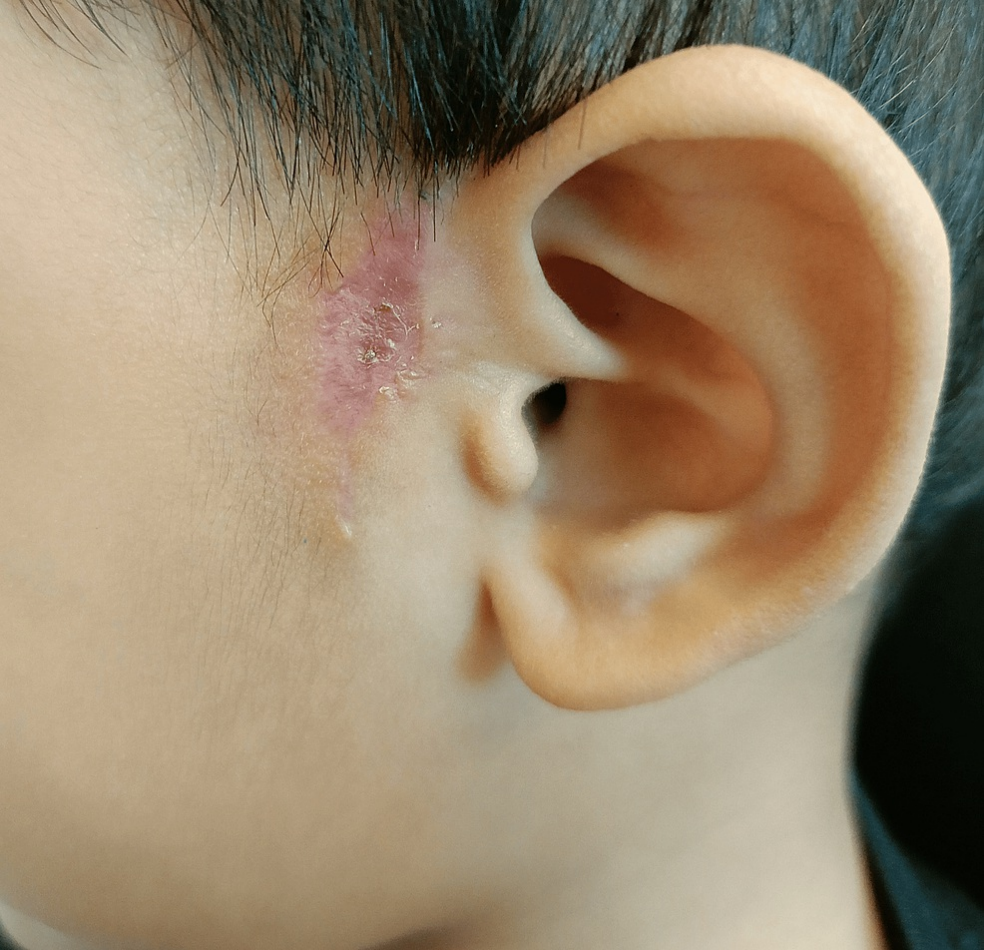
Well-healed wound at three months post-excision with no evidence of recurrence

Careful planning for wound closure was undertaken, anticipating a large skin defect following the excision. Considering the patient's clinical presentation, tissue viability, and potential for wound healing, we opted for wide-undermining, followed by subcutaneous closure using Vicryl 4-0 suture, and subsequent Surgiseal® application (Figures 2-3). Meticulous surgical technique is essential in achieving complete excision and successful wound closure. The elliptical incision, inclusive of the sinus opening and scar tissue, was made with a superior extension to the supraauricular region. The incision was deepened till the temporalis fascia, which forms the medial limit of the dissection. The subcutaneous tissue present between the temporalis fascia and the helix perichondrium was removed along with a small portion of the helical cartilage at the apex of the sinus tract. The child was prescribed a one-week course of oral amoxicillin-clavulanic acid. The post-operative period was uneventful, with no evidence of wound dehiscence or infection. At the three-month post-operative review, the wound had healed well with satisfactory cosmesis (Figure 4), and the parents were content with the outcome.

## Discussion

Symptomatic preauricular sinuses require excision as definitive management. The aims of preauricular sinus excision are, firstly, to identify the sinus and all its ramifications, followed by complete excision, and, secondly, to minimize any potential cosmetic defect. However, sinuses that have been recurrently infected and undergone repeated drainage may present a challenging entity for surgeons, in ensuring complete excision with a successful outcome. Moreover, in cases with involvement of the preauricular skin, the surgery may necessitate wide local excision of the overlying skin. Hence, surgeons are faced with reconstructive challenges to ensure a balance between the optimization of functional and aesthetic outcomes. Since preauricular sinus excision involves facial surgical defects, the emphasis on aesthetic outcomes should not be overlooked.

The surgical techniques employed to excise the preauricular sinus have been discussed widely in the literature. They can be broadly divided into the classic simple sinusectomy and the supraauricular approach [4]. Various technical modifications have been further introduced to maximize surgical outcomes. Among these modifications are the use of a lacrimal probe, methylene blue staining, and the use of an operating microscope or surgical loupes. These improvisations are mainly targeted at identifying the ramifications and full extent of the preauricular sinuses [5].

It is well-accepted that the recurrence rate of preauricular sinuses is higher in cases with prior infection, and even more so with prior incision and drainage. Post-infection inflammation and scarring lead to fibrosis that complicates the complete excision of the sinus tract. Hence, recurrence occurs when residual squamous epithelium is left behind [6]. This was a pertinent concern in our patient, as he has had multiple episodes of infection as well as drainage procedures. In fact, the complexity of this case was accentuated by the thinning of the preauricular skin, with visible pulsations of the superficial temporal artery (STA). It is worth mentioning the high risk of arterial injury, not just intraoperatively, but also due to any forms of facial trauma that may possibly occur [7].

The techniques for wound closure depend primarily on the depth of the defect and the degree of tension. Lee et al. proposed a reconstruction algorithm for wound defects post-preauricular sinus excision based on the earlier-mentioned factors. The techniques employed were either simple sutures, primary repair with wide-undermining, or the use of a posterior auricular flap [8]. For our patient, we anticipated a large wound defect and difficult primary closure. Factors such as young age, cosmesis, risk of wound dehiscence, and need for subsequent repair were considered before arriving at the final approach. We opted for primary repair with wide-undermining, in which subcutaneous closure was followed by the application of topical skin adhesive, which provided further tissue strength besides acting as an additional barrier against infection. The waterproof property of Surgiseal® allows the child to have brief water exposure without risking wound integrity. This technique also eliminated the need for suture removal, which could be distressing in pediatric patients, besides reducing the risk of foreign body reactions associated with sutures. Topical tissue adhesives, particularly OCA, have gained a stronghold as a safe and effective method when used correctly on appropriately selected wounds [9]. Caution has to be taken to apply it only on the skin, as contact with subdermal layers may elicit foreign body reactions, leading to chronic pain or inflammation.

## Conclusions

Complicated preauricular sinuses with recurrent infections can result in significant skin scarring, necessitating wide local excision as the definitive treatment. However, this approach often poses a reconstructive challenge due to the resultant sizable defect. Our experience with this case provides valuable insight into the management of complicated preauricular sinuses, demonstrating the feasibility of primary closure even in such challenging scenarios. The use of Surgiseal® underscores its role in facilitating successful wound closure with favorable cosmetic results. Ongoing research in the field of tissue adhesives aims to create the ideal material, characterized by excellent healing properties, good cosmetic outcomes, and minimal immunological reactions. Emphasizing prompt surgical intervention for infected sinuses is critical to optimizing postoperative outcomes and minimizing complications.
